# 3D-QSAR and Molecular Docking Studies on Fused Pyrazoles as p38α Mitogen-Activated Protein Kinase Inhibitors

**DOI:** 10.3390/ijms11093357

**Published:** 2010-09-17

**Authors:** Ping Lan, Zhi-Jian Huang, Jun-Rong Sun, Wei-Min Chen

**Affiliations:** Guangdong Province Key Laboratory of Pharmacodynamic Constituents of TCM and New Drugs Research, College of Pharmacy, Jinan University, Guangzhou 510632, Guangdong, China; E-Mails: lanpingsmzh@126.com (P.L.); huangzhijian0505@gmail.com (Z.-J.H.); sunjunrong1986@yahoo.com.cn (J.-R.S.)

**Keywords:** p38α mitogen-activated protein kinase, 3D-QSAR, CoMFA, CoMSIA, docking

## Abstract

The p38α mitogen-activated protein kinase (MAPK) has become an attractive target for the treatment of many diseases such as rheumatoid arthritis, inflammatory bowel disease and Crohn’s disease. In this paper, 3D-QSAR and molecular docking studies were performed on 59 p38α MAPK inhibitors. Comparative molecular field analysis (CoMFA) and comparative molecular similarity indices analysis (CoMSIA) were applied to determine the structural requirements for potency in inhibiting p38α MAPK. The resulting model of CoMFA and CoMSIA exhibited good *r*^2^_cv_ values of 0.725 and 0.609, and *r^2^* values of 0.961 and 0.905, respectively. Molecular docking was used to explore the binding mode between the inhibitors and p38α MAPK. We have accordingly designed a series of novel p38α MAPK inhibitors by utilizing the structure-activity relationship (SAR) results revealed in the present study, which were predicted with excellent potencies in the developed models. The results provided a useful guide to design new compounds for p38α MAPK inhibitors.

## 1. Introduction

The p38 mitogen-activated protein kinase (MAPK), a serine/threonine kinase, plays a crucial role in biosynthesis of pro-inflammatory cytokines including tumor necrosis factor α (TNFα) and interleukin-1 β (IL-1β) [1]. The p38 MAPK exists in four isoforms (α, β, γ and δ). Expression of these isoforms varies across cell types of the immune system, and it was revealed by previous studies that the predominant isoform involved in inflammation is p38α [2,3]. The p38α MAPK is activated by a range of environmental stimuli such as TNFα, IL-1β and stress. Activation of p38α leads to the up-regulation of both TNFα and IL-1β, resulting in many chronic inflammatory diseases, such as rheumatoid arthritis (RA), inflammatory bowel disease, Crohn’s disease, and chronic obstructive pulmonary disease (COPD), as well as the other inflammatory disorders [4–8].

The proven ability of p38 MAPK to efficiently regulate both the release and the activity of these pro-inflammatory cytokines has prompted many pharmaceutical groups to pursue inhibitors of p38 MAPK for the potential treatment of various inflammatory diseases [9–11]. A series of fused pyrazole derivatives with potent, selective and orally available inhibition activities towards p38 MAPK were reported [1–3]. In this paper, 3D-QSAR and molecular docking studies were performed on these inhibitors. Along with the molecular docking, 3D-QSAR approaches including comparative molecular field analysis (CoMFA) and comparative molecular similarity analysis (CoMSIA) could offer more insight into understanding the structure-activity relationship of these inhibitors, and thus could more effectively direct the design of new potential inhibitors. Based on the structure-activity relationship revealed in the present study, a series of novel p38α MAPK inhibitors were accordingly designed.

## 2. Materials and Methods

### 2.1. Dataset for Analysis

All the compounds and associated biological activities were selected from literature [1–3] reported by the same research group. These fused pyrazole derivatives were divided into a training set of 46 compounds and a test set of 13 compounds. The inhibitory data was reported as IC_50_ towards p38α MAPK, and the IC_50_ values were converted into corresponding pIC_50_ by taking Log (1/IC_50_). The pIC_50_ values were used as the dependent variables in all the models subsequently developed. Structures and associated inhibitory activities are shown in Tables 1 and 2.

### 2.2. Molecular Modeling and Alignment

**Figure 1:**
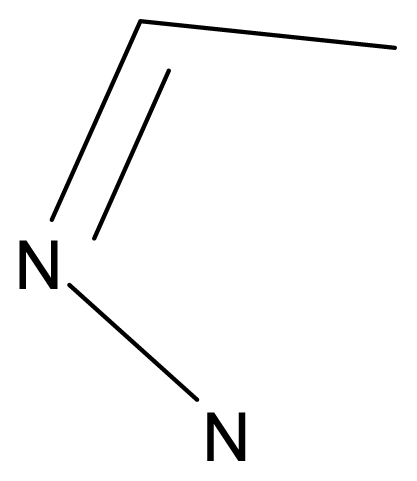
Fragment used as a common structure in the alignments.

**Figure 2:**
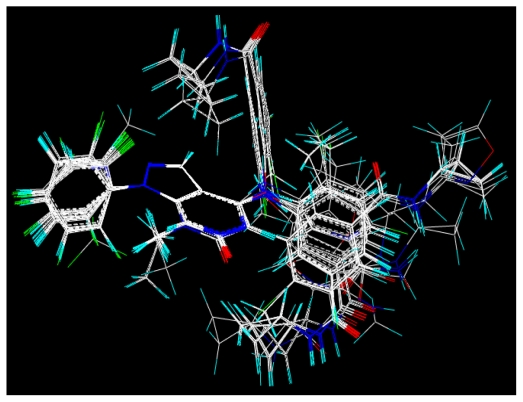
Alignment of the molecules used in the training set.

All computational studies were performed using SYBYL 8.1 molecular modeling software from Tripos, Inc., US [12]. 3D structures of all compounds were constructed using the Sketch Molecule module. Structural energy minimization was performed using the standard Tripos molecular mechanics force field and Gasteiger-Hückel charge [13]. The fragment that was used as the common structure is shown in Figure 1. The compound **9** was selected as a template molecule, which was one of the most active derivatives in the dataset. The aligned molecules of the training set are shown in Figure 2.

### 2.3. CoMFA and CoMSIA Setup

To calculate the CoMFA and CoMSIA fields, a 3D cubic lattice with grid spacing of 1 Å and extending to 4 Å units beyond the aligned molecules in all directions was created automatically by SYBYL [12]. In the CoMFA method, a *sp*^3^ hybridized carbon atom with a charge of 1e served as the probe atom to calculate steric and electrostatic fields, in which their energy values were truncated at 30 kcal/mol [14]. The CoMSIA method, incorporating steric, electrostatic, hydrophobic, hydrogen bond donor and acceptor fields, was carried out using a probe atom with radius 1.0 Å, +1.0 charge, and hydrophobic and hydrogen bond properties of +1. The attenuation factor was set to the default value of 0.3 [15].

### 2.4. Regression Analysis and Models Validation

Partial Least Squares (PLS) was used to linearly correlate the CoMFA and CoMSIA fields to the pIC_50_ values. The performance of both the CoMFA and CoMSIA models was evaluated using the leave-one-out (LOO) method [16]. PLS was conjunct with the cross-validation option to determine the optimum number of components (ONC), which were then used in deriving the final CoMFA and CoMSIA model without cross-validation. The ONC was the number of components resulting in the highest cross-validated correlation coefficient (*r*^2^_cv_). After obtaining the optimum number of components, a PLS analysis was performed with no validation and column filtering 2.0 to generate the highest correlation coefficient (*r*^2^) [17–19].

### 2.5. Predictive Correlation Co-Efficient (*r*^2^_pred_)

The predictive abilities were determined from a test set of 13 compounds that were not included in the training set. These molecules were aligned to the template and their pIC_50_ values were predicted. The predictive correlation coefficient (*r*^2^_pred_), based on the molecules of test set, was calculated by the equation shown below.

$${r^{2}}_{\text{pred}}=(\text{SD}-\text{PRESS})/\text{SD}$$

SD is the sum of the squared deviations between the inhibitory activities of the test set and mean activities of the training molecules and PRESS is the sum of squared deviations between predicted and actual activity values for each molecule in the test set [20–22].

### 2.6. Molecular Docking

To investigate the protein-ligand interactions, compound **9** was docked into the ATP-binding site of p38α MAPK. The Surflex-Dock, using an empirical scoring function and a patented search engine to dock ligands into a protein’s binding site, was applied to study molecular docking [12]. The crystal structure of p38α MAPK was retrieved from the RCSB Protein Data Bank (PDB entry code: 3LHJ). The p38α MAPK structure was utilized in subsequent docking experiments without energy minimization. The ligands were docked into the corresponding protein’s binding site by an empirical scoring function and a patented search engine in Surflex-Dock [12]. All ligands and water molecules were removed and the polar hydrogen atoms were added. Protomol, an idealized representation of a ligand that makes every potential interaction with the binding site, was used to guide molecular docking. The establishment of protomol demonstrates three behaviors: (a) Automatic: Surflex-Dock finds the largest cavity in the receptor protein; (b) Ligand: a ligand in the same coordinate space as the receptor; (c) Residues: specified residues in the receptor [10]. In this paper, the automatic docking was applied. To visualize the binding mode between the protein and ligand, the MOLCAD (Molecular Computer Aided Design) program was employed. MOLCAD calculates and displays the surfaces of channels and cavities, as well as the separating surface between protein subunits [12]. The MOLCAD program provides a variety of properties and maps onto protein surfaces. In this paper, the cavity depth, electrostatic and lipophilic potential surfaces were established by MOLCAD. Other parameters were established by default in the software.

## 3. Results and Discussion

### 3.1. CoMFA and CoMSIA Analysis

**Figure 3:**
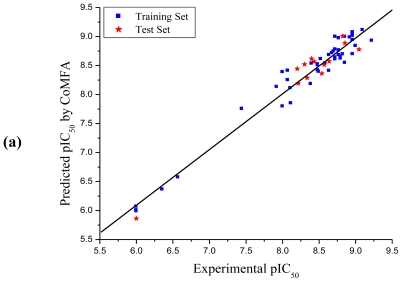

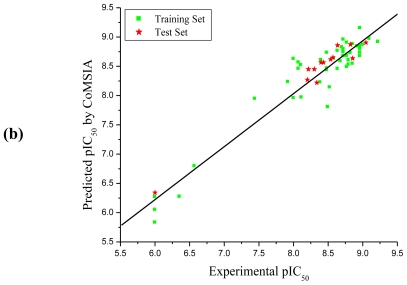
Graph of experimental *versus* predicted pIC_50_ of the training set and the test set using CoMFA (**a**) and CoMSIA (**b**).

The statistical parameters obtained from the CoMFA model are listed in Table 3. The CoMFA model, using 46 and 13 derivatives in the training and test sets, respectively, gave a cross-validated correlation coefficient (*r^2^_cv_*) of 0.725 (>0.6) with an optimized component of 6, which suggests that the model should be a useful tool for predicting the IC_50_ values. An excellent non-cross-validated correlation coefficient (*r^2^*) of 0.961 with a low standard error estimate (SEE) of 0.178, *F* value of 160.943 and predictive correlation coefficient (*r*^2^_pred_) of 0.953 was obtained. Contributions of steric and electrostatic fields were 0.534 and 0.466, respectively. The CoMSIA model, using steric, electrostatic, hydrophobic, hydrogen bond donor and hydrogen bond acceptor fields, gave a good cross-validated correlation coefficient (*r^2^_cv_*) of 0.609 (>0.6) with an optimized component of 6. A high non-cross-validated correlation coefficient (*r^2^*) of 0.905 with a SEE of 0.279, *F* value of 61.672 and predictive correlation coefficient (*r*^2^_pred_) of 0.929 was obtained. Contributions of steric, electrostatic, hydrophobic, hydrogen bond donor and hydrogen bond acceptor fields were 0.159, 0.137, 0.202, 0.310 and 0.192, respectively. The experimental and predicted pIC_50_ values and residual values for the training set and test set compounds in CoMFA and CoMSIA are given in Table 2. The relationship between experimental and predicted pIC_50_ values of the training set and test set compounds in CoMFA and CoMSIA are illustrated in Figure 3(a),(b).

### 3.2. Graphical Interpretation of CoMFA and CoMSIA

**Figure 4:**
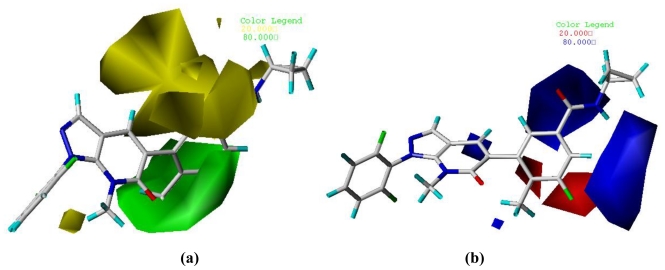
CoMFA Std* coeff contour maps illustrating steric, electrostatic fields in combination with compound **9**. (**a**) Steric fields: green contours (80% contribution) indicate regions where bulky groups increase activity, while yellow contours (20% contribution) indicate regions where bulky groups decrease activity, and (**b**) Electrostatic fields: blue contours (80% contribution) indicate regions where electron-donating groups increase activity, while red contours (20% contribution) indicate regions where electron-withdrawing groups increase activity.

One of the attractive features of the CoMFA and CoMSIA models is the visualization of the results as 3D coefficient contour maps. To visualize the information content of the derived 3D-QSAR model, CoMFA and CoMSIA contour maps were generated to rationalize the regions in 3D space around the molecules where changes in each field were predicted to increase or decrease the activity. The CoMFA steric and electrostatic contour maps that are shown in Figure 4 use compound **9** as a reference structure. In Figure 4(a), the green contours represent regions of high steric tolerance (80% contribution) while the yellow contours represent regions of low steric bulk tolerance (20% contribution). In Figure 4(b), the electrostatic field is indicated by blue (80% contribution) and red (20% contribution) contours, which reveal the regions where electron-donating group and electron-withdrawing group would be favorable, respectively.

As shown in Figure 4(a), the yellow contour near R_1_ position indicates that bulky groups would decrease the potency. Comparing compound **27** with **24**, their activity discrepancies can be explained by this yellow contour. A huge green contour around the R_3_ and R_4_ position suggested that bulkier groups would be favored. Most of the derivatives possessed a relatively bulkier methyl substituent at the R_3_ position, compounds **17–19,** which had minor groups (e.g., H, F), showed significantly decreased activities. In Figure 4(b), the blue contour near the R_3_ position indicates that electron-donating groups may increase the activity. This may explain why compounds **17–19**, without an electron-donating substituent at this position, were the most inactive derivatives. A red contour near the R_4_ position demonstrated that electron-withdrawing groups would benefit the activity, compound **9** with an electron-withdrawing substituent (-F) at R_4_ showed significantly increased activity. Two blue contours around the R_5_ position strongly revealed that an electron-donating group would be favorable. Most of the compounds in the database possessed an electron-donating cyclopropyl substituent at R_5_, compounds **17–22** and **26—**without an electron-donating group at this position—showed significantly decreased activities.

**Figure 5:**
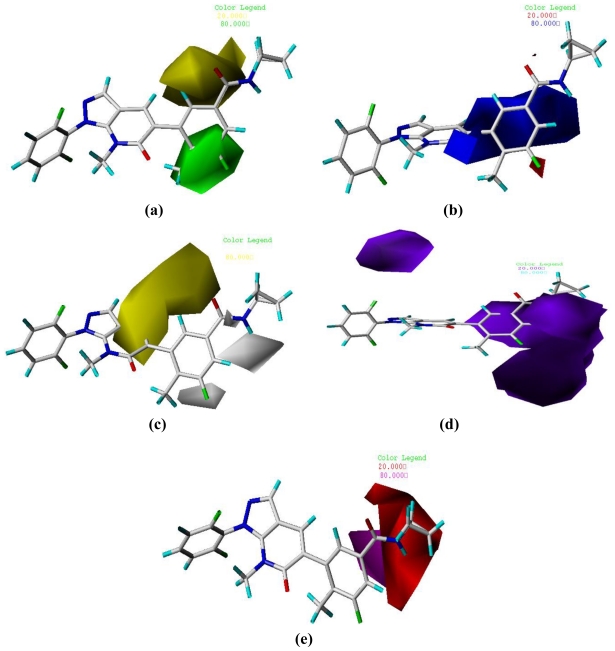
Std* coeff contour maps of CoMSIA illustrating steric, electrostatic, hydrophobic, hydrogen bond donor and acceptor fields in combination with compound **9**. (**a**) Steric contour map. Green contours refer to sterically favored regions while yellow contours refer to sterically disfavored regions. (**b**) Electrostatic contour map. Blue contours refer to regions where electron-donating groups are favored; red contours indicate regions where electron-withdrawing groups are favored. (**c**) Hydrophobic contour map. White contours (80% contribution) refer to regions where hydrophilic substituents are favored; yellow contours (20% contribution) indicate regions where hydrophobic substituents are favored. (**d**) Hydrogen bond donor contour map. The cyan (80% contribution) and purple (20% contribution) contours indicate favorable and unfavorable hydrogen bond donor groups. (**e**) Hydrogen bond acceptor contour map. The purple contours (80% contribution) for hydrogen bond acceptor groups increase activity, red contours (20% contribution) indicate the disfavored region.

The CoMSIA steric, electrostatic, hydrophobic, hydrogen bond donor and acceptor field contour maps are shown in Figure 5 using compound **9** as a reference structure. The CoMSIA steric and electrostatic field contour maps were almost similar to the corresponding CoMFA contour maps.

The hydrophobic field contour map is shown in Figure 5(c), white (20% contribution) and yellow (80% contribution) contours highlight areas where hydrophilic and hydrophobic properties were favored. The yellow contour around the C-6 position revealed the extreme importance of the phenyl group at this position; replacing it with a hydrophilic group may result in decreased activity. Two white contours near R_4_ and N-15 positions indicated that hydrophilic substituents may be favored.

The hydrogen bond donor field contour map is illustrated in Figure 5(d), the cyan (80% contribution) and purple (20% contribution) contours indicate favorable and unfavorable hydrogen bond donor groups. The purple contour near the C′-2 position of the phenyl group in R_2_ indicates that a hydrogen bond donor would be unfavorable. In general, compounds with a hydrogen bond acceptor substituent (e.g., -F) at the C′-2 position showed better activities than those without a hydrogen bond acceptor group. The carbonyl group in the C-14 position was surrounded by a huge purple contour, suggesting it may serve as a hydrogen bond acceptor and that removing it may result in decreased potency.

The hydrogen bond acceptor field contour map is depicted in Figure 5(e), the purple (80% contribution) and red (20% contribution) contours favorable and unfavorable positions for hydrogen bond acceptors. The purple contour near the carbonyl group in the C-14 position revealed a hydrogen bond acceptor substituent at this position would benefit the activity. A red contour near N-15 position revealed the importance of the hydrogen bond donor -NH group.

### 3.3. Docking Analysis

**Figure 6:**
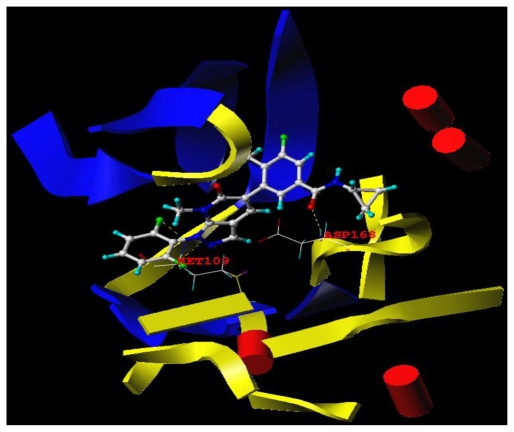
MOLCAD Robbin surfaces structure of selected compound **9** in complex with the ATP pocket of P38α MAPK (PDB code: 3LHJ). Key residues and hydrogen bonds are labeled. The alpha helices are shown as yellow helices or cylinders, while beta sheets are shown as blue arrows and the loop regions as red tubes.

To explore the interaction mechanism between these inhibitors and the receptor, compound **9** was selected for more detailed analysis. The MOLCAD Robbin surfaces structure of the ATP pocket of the p38α MAP within the compound **9** is shown in Figure 6. The -F at C′-2 position acted as a hydrogen bond acceptor and formed a H-bond with the -NH of the Met 109 residue, the carbonyl group at C-14 position also acted as a hydrogen bond acceptor by forming a H-bond with the -NH of the ASP168. The observations obtained from Figure 6 are in agreement with that of CoMSIA hydrogen bond donor and acceptor contour maps.

The MOLCAD Robbin and Multi-Channel surfaces structure of the ATP-binding site of the p38α MAP were also developed and displayed with cavity depth, electrostatic potential as well as lipophilic potential to explore the ligand-receptor interactions, furthermore, to examine the 3D contour maps obtained by CoMFA and CoMSIA.

**Figure 7:**
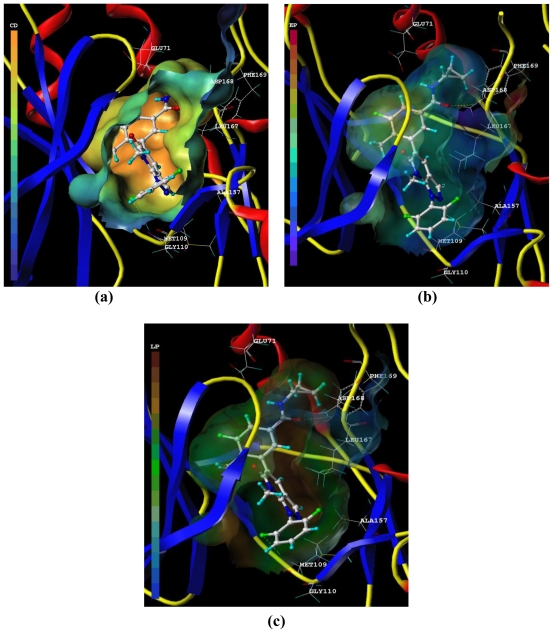
The MOLCAD Robbin and Multi-Channel surfaces structure displayed with cavity depth (**a**), electrostatic (**b**) and lipophilic (**c**) potential surfaces of the ATP pocket of p38α MAPK within the compound **9**. The cavity depth color ramp ranges from blue (low depth values = outside of the pocket) to light red (high depth values = cavities deep inside the pocket). The color ramp for EP ranges from red (most positive) to purple (most negative). The color ramp for LP ranges from brown (highest lipophilic area of the surface) to blue (highest hydrophilic area).

Figure 7(a) depicts the MOLCAD cavity depth potential surface of the ATP pocket within compound **9**, the cavity depth color ramp ranges from blue (low depth values = outside of the pocket) to light red (high depth values = cavities deep inside the pocket). As shown in Figure 7(a), the phenyl group of R_2_ position was in the relative lower depth, while the other parts of the compound **9** were anchored deep inside the ATP pocket.

Figure 7(b) demonstrates the MOLCAD electrostatic potential surface of the ATP-binding region, the color ramp for EP ranges from red (most positive) to purple (most negative). In Figure 7(b), the R_3_ position was found in a yellow area, which indicated that electron-withdrawing properties were essential for potency; the R_5_ position was in a blue area, which suggested that electron-donating properties were crucial for activity. The observations obtained from this electrostatic potential surface were satisfied according to the corresponding CoMFA and CoMSIA electrostatic contour maps.

Figure 7(c) shows the MOLCAD lipophilic potential surface of the ATP pocket, the color ramp for LP ranges from brown (highest lipophilic area of the surface) to blue (highest hydrophilic area). The phenyl group at C-5 position was in a brown area, which revealed the importance of hydrophobic properties for this position; the N-15 position was in a white area, which indicated the significance of hydrophilic properties for this position. These observations are in agreement with that obtained from the CoMSIA hydrophobic contour map.

### 3.4. Summary of the Structure-Activity Relationship

**Figure 8:**
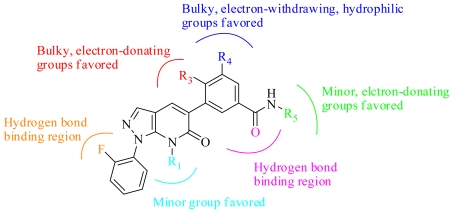
Summary of structure-activity relationship revealed by 3D-QSAR and docking.

The structure-activity relationship revealed by 3D-QSAR and molecular docking studies is illustrated in Figure 8. In detail, the minor substituent in the R_1_ position would be favored; the bulky, electron-donating groups in R_3_ position would increase the activity; the bulky, electron-withdrawing, hydrophilic groups in R_4_ position would benefit the potency; the minor, electron-donating substituent in R_5_ position would be favorable; the -F at the C′-2 position and the carbonyl group at C-14 position are crucial for binding to the ATP pocket.

### 3.5. Design for New Inhibitors

**Figure 9:**
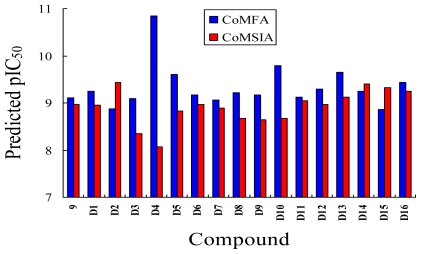
Graph of the predicted pIC_50_ values of the designed inhibitors using CoMFA and CoMSIA.

Based on the structure-activity relationship revealed by this study, we have designed a series of novel inhibitors, these molecules were aligned to the database and their activities were predicted by the CoMFA and CoMSIA models previously established. The chemical structures and predicted pIC_50_ values of these compounds are shown in Table 4. The predicted pIC_50_ values of these compounds *versus* that of the most active compound **9** are illustrated in Figure 9. Most of the designed molecules showed better pIC_50_ values than compound **9**, which validated the structure-activity relationship obtained in this study.

## 4. Conclusion

In conclusion, the 3D-QSAR and docking models established in our present study are quite reliable to efficiently guide further modification of the fused pyrazole derivatives for obtaining better inhibitors. Both the CoMFA and CoMSIA models provided the significant correlations of biological activities with steric, electrostatic, hydrophobic, hydrogen bond donor and acceptor fields. In comparison to CoMSIA, the CoMFA method was found to afford a slightly better predictability. The structure-activity relationships revealed by 3D-QSAR and docking were validated by newly designed derivatives. The results served as a useful guideline for developing novel p38α MAPK inhibitors.

## Figures and Tables

**Table 1:** The chemical structures of the fused pyrazole derivatives used for the training and test set.

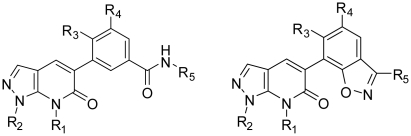					
Compound No.	Substituent				
	R_1_	R_2_	R_3_	R_4_	R_5_
**1**	Me	2-Me-Ph	Me	H	Cyclopropyl
**2**	Me	2-Cl-Ph	Me	H	Cyclopropyl
**3**	Me	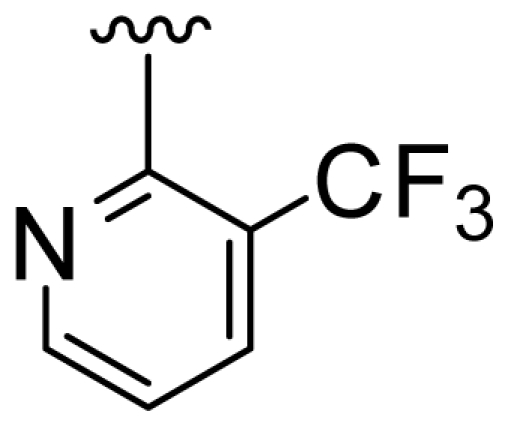	Me	H	Cyclopropyl
**4**	Me	2,4-Di-F-Ph	Me	H	Cyclopropyl
**5**	Me	2,5-Di-F-Ph	Me	H	Cyclopropyl
**6**	Me	2,6-Di-F-Ph	Me	H	Cyclopropyl
**7**	Et	2,4-Di-F-Ph	Me	H	Cyclopropyl
**8**	Me	2,6-Di-F-Ph	Cl	H	Cyclopropyl
**9**	Me	2,6-Di-F-Ph	Me	F	Cyclopropyl
**10**	Me	2,6-Di-F-Ph	Me	H	H
**11**	Me	2,6-Di-F-Ph	Me	H	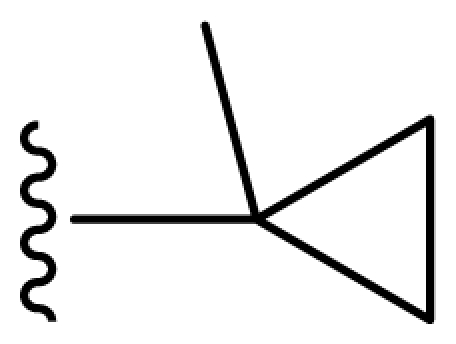
**12**	Me	2,6-Di-F-Ph	Me	H	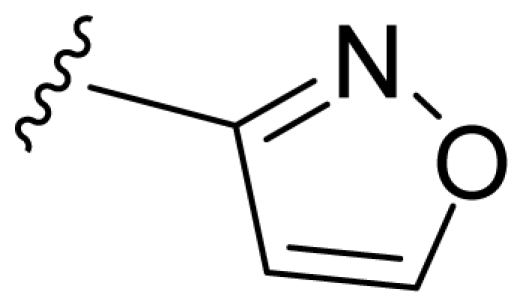
**13**	Me	2,6-Di-F-Ph	Me	H	Methylamino

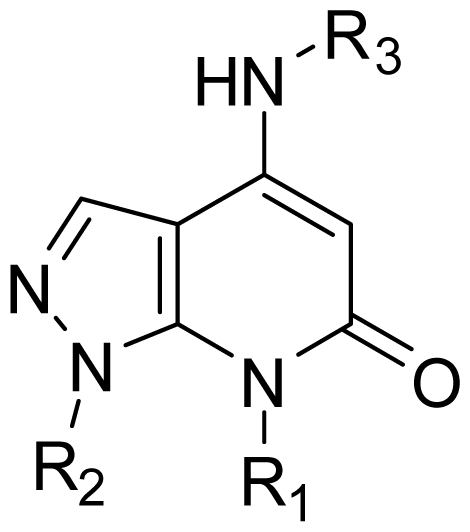			
Compound No.	Substituent		
	R_1_	R_2_	R_3_
**14**	Me	3-F-Ph	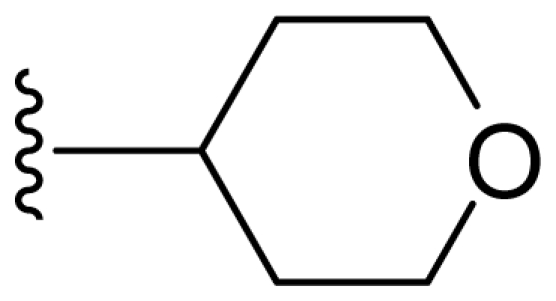
**15**	Me	2,6-Di-F-Ph	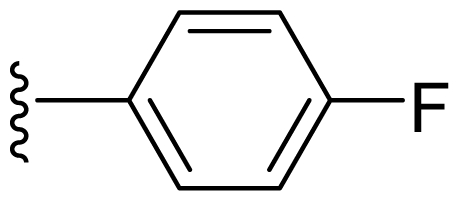
**16**	Me	2,4-Di-F-Ph	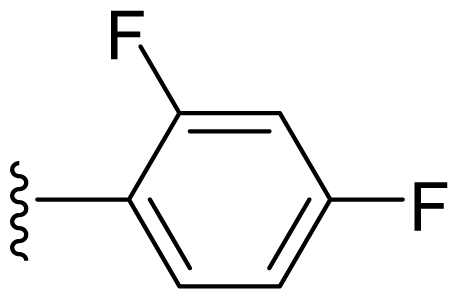
**17**	Me	2,5-Di-F-Ph	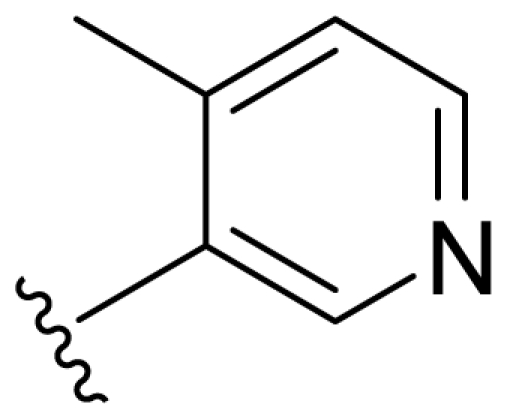

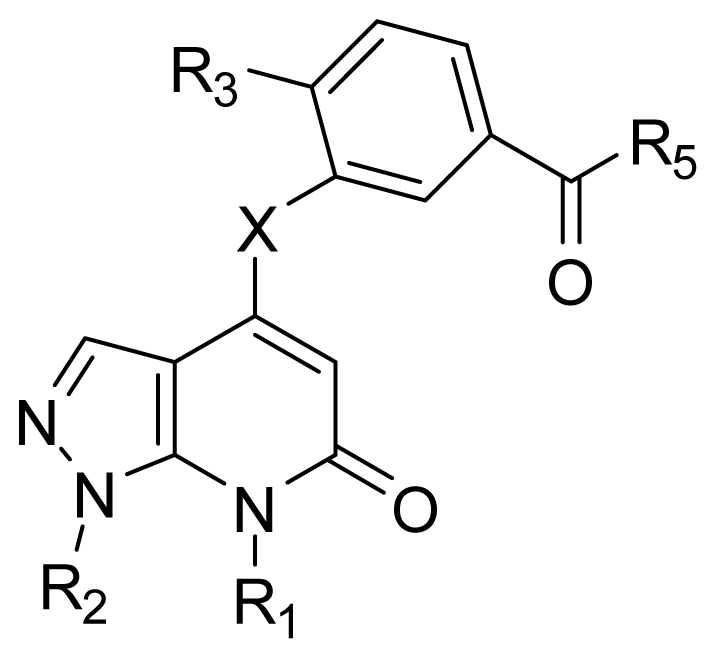					
Compound No.	Substituent				
	R_1_	R_2_	X	R_3_	R_5_
**18**	Me	2,4-Di-F-Ph	NH	Me	NH_2_
**19**	Me	2,5-Di-F-Ph	NH	Me	NH_2_
**20**	Me	2,4-Di-F-Ph	NH	Me	NHOMe
**21**	Me	2,4-Di-F-Ph	NH	Me	Cyclopropylamino
**22**	Me	2,4-Di-F-Ph	NH	Me	t-butylamino
**23**	Me	2,4-Di-F-Ph	NH	Me	OH
**24**	Me	3-F-Ph	NH	Me	Cyclopropylamino
**25**	Me	3-F-Ph	O	Me	Cyclopropylamino
**26**	Me	4-F-Ph	NH	Cl	Cyclopropylamino
**27**	Et	4-F-Ph	NH	Me	Cyclopropylamino
**28**	Et	4-F-Ph	NH	Cl	Cyclopropylamino
**29**	Et	4-F-Ph	O	Me	Cyclopropylamino
**30**	Et	2,4-Di-F-Ph	NH	Cl	Cyclopropylamino
**31**	Me	2,4-Di-F-Ph	O	Me	Cyclopropylamino
**32**	Me	2,5-Di-F-Ph	NH	Me	Cyclopropylamino
**33**	Me	2,5-Di-F-Ph	NH	Cl	Cyclopropylamino
**34**	Me	2,5-Di-F-Ph	O	Me	Cyclopropylamino
**35**	Me	2,5-Di-F-Ph	O	Cl	Cyclopropylamino
**36**	Me	2,6-Di-F-Ph	NH	Me	Cyclopropylamino
**37**	Me	2,6-Di-F-Ph	NH	Cl	Cyclopropylamino
**38**	Me	2,6-Di-F-Ph	NH	F	Cyclopropylamino
**39**	Me	2,6-Di-F-Ph	O	Me	Cyclopropylamino

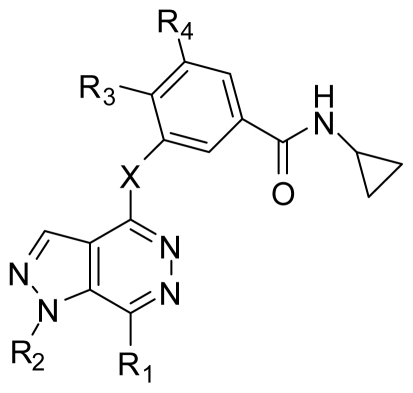					
Compound No.	Substituent				
	R_1_	R_2_	X	R_3_	R_4_
**40**	H	2-Cl-Ph	NH	Cl	H
**41**	H	3-F-Ph	NH	Me	H
**42**	H	3-F-Ph	NH	Cl	H
**43**	H	4-F-Ph	NH	Cl	H
**44**	H	2,4-Di-F-Ph	NH	Me	H
**45**	H	2,4-Di-F-Ph	NH	Cl	H
**46**	H	2,4-Di-F-Ph	O	Me	H
**47**	H	2,4-Di-F-Ph	O	Cl	H
**48**	H	2,5-Di-F-Ph	NH	Cl	H
**49**	H	2,6-Di-F-Ph	NH	Me	H
**50**	H	2,6-Di-F-Ph	NH	Cl	H
**51**	H	2,6-Di-F-Ph	O	Me	H
**52**	Me	4-F-Ph	NH	Me	H
**53**	Me	4-F-Ph	NH	Cl	H
**54**	Me	4-F-Ph	O	Me	H
**55**	Me	2,4-Di-F-Ph	NH	Me	H
**56**	Me	2,4-Di-F-Ph	NH	Me	F
**57**	Me	2,4-Di-F-Ph	NH	Cl	H
**58**	Me	2,4-Di-F-Ph	O	Me	H
**59**	Me	2,6-Di-F-Ph	NH	Cl	H

**Table 2:** The experimental pIC_50_ values, predicted pIC_50_ values (Pred.) and their residuals (Res.) of the pyrazole derivative training and test set molecules.

Compd. No.	Experimental	CoMFA		CoMSIA	

		Pred.	Res.	Pred.	Res.
**1**	8.721	8.777	−0.056	8.957	−0.236
2*	9.046	8.778	0.268	8.905	0.141
**3**	8.638	8.682	−0.044	8.764	−0.126
4*	8.638	8.572	0.066	8.858	−0.220
**5**	8.721	8.639	0.082	8.790	−0.069
**6**	8.854	8.995	−0.141	8.879	−0.025
**7**	9.222	8.929	0.293	8.920	0.302
**8**	8.959	9.010	−0.051	8.841	0.118
**9**	9.097	9.111	−0.014	8.972	0.125
10*	8.824	9.006	−0.182	8.873	−0.049
**11**	8.770	8.972	−0.202	8.903	−0.133
**12**	9.000	8.839	0.161	8.868	0.132
**13**	8.699	8.742	−0.043	8.826	−0.127
**14**	6.000	6.004	−0.004	5.830	0.170
**15**	6.000	6.067	−0.067	6.266	−0.266
16*	6.000	5.862	0.138	6.340	−0.340
**17**	6.353	6.362	−0.009	6.273	0.080
**18**	8.585	8.533	0.052	8.637	−0.052
19*	8.432	8.568	−0.136	8.566	−0.134
**20**	8.959	9.046	−0.087	9.152	−0.193
**21**	8.495	8.396	0.099	7.804	0.691
**22**	6.570	6.573	−0.003	6.795	−0.225
**23**	6.000	5.986	0.014	6.047	−0.047
**24**	8.482	8.521	−0.040	8.435	0.047
**25**	8.071	8.248	−0.177	8.566	−0.495
**26**	8.824	8.695	0.129	8.728	0.096
27*	8.398	8.617	−0.219	8.565	−0.167
**28**	8.921	8.985	−0.064	8.840	0.081
**29**	8.398	8.533	−0.135	8.608	−0.210
**30**	8.959	9.075	−0.116	8.800	0.159
**31**	8.108	8.106	0.002	8.519	−0.411
32*	8.538	8.362	0.176	8.617	−0.079
**33**	8.959	8.696	0.263	8.676	0.283
34*	8.215	8.192	0.023	8.449	−0.234
**35**	8.482	8.504	−0.022	8.732	−0.250
**36**	8.721	8.606	0.115	8.674	0.047
**37**	8.959	8.939	0.020	8.735	0.224
**38**	8.721	8.996	−0.275	8.744	−0.023
**39**	8.854	8.546	0.308	8.543	0.311
**40**	8.796	8.622	0.174	8.529	0.267
41*	8.337	8.287	0.050	8.220	0.117
42*	8.301	8.519	−0.218	8.450	−0.149
**43**	8.071	8.410	−0.339	8.459	−0.388
**44**	7.921	8.132	−0.211	8.232	−0.311
**45**	8.482	8.419	0.063	8.463	0.019
**46**	8.000	7.795	0.205	7.956	0.044
**47**	7.444	7.754	−0.310	7.946	−0.502
**48**	8.678	8.714	−0.036	8.588	0.090
**49**	8.770	8.761	0.009	8.680	0.090
50*	8.854	8.887	−0.033	8.635	0.219
**51**	8.523	8.612	−0.089	8.140	0.383
**52**	8.387	8.182	0.205	8.225	0.162
**53**	8.638	8.409	0.229	8.457	0.181
**54**	8.114	7.849	0.265	7.968	0.146
55*	8.569	8.513	0.056	8.649	−0.080
**56**	8.000	8.387	−0.387	8.627	−0.627
**57**	8.796	8.648	0.148	8.605	0.191
58*	8.201	8.444	−0.243	8.269	−0.068
**59**	8.770	8.681	0.089	8.489	0.281

* Test set molecules.

**Table 3:** The partial least squares (PLS) results obtained using the CoMFA and CoMSIA models.

PLS Statistics	CoMFA	CoMSIA
*r*^2^_cv_^a^	0.725	0.609
*r*^2^^b^	0.961	0.905
ONC^c^	6	6
SEE^d^	0.178	0.279
F value^e^	160.943	61.672
*r*^2^_pred_^f^	0.953	0.929
Field contribution		
Steric	0.534	0.159
Electrostatic	0.466	0.137
Hydrophobic	-	0.202
H-bond Donor	-	0.310
H-bond Acceptor	-	0.192

a cross-validated correlation coefficient;

b non-cross-validated coefficient;

c optimal number of components;

d standard error of estimate;

e F-test value;

f predictive correlation coefficient.

**Table 4:** Chemical structures of the newly designed inhibitors and their pIC_50_ values.

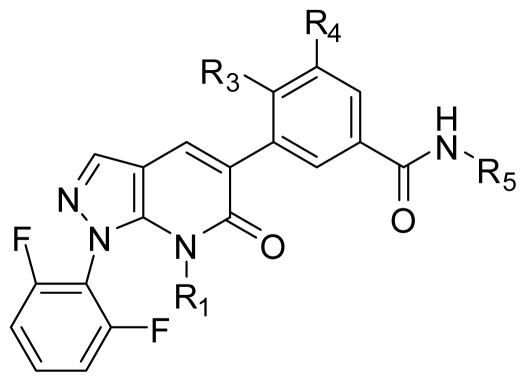						
No.	Substituent			Predicted pIC_50_		

	R_1_	R_3_	R_4_	R_5_	CoMFA	CoMSIA
**D1**	Me	CH_2_OOCCH_3_	COOH	Et	9.253	8.961
**D2**	Me	CH_2_OOCCH_3_	CONH_2_	Et	8.881	9.438
**D3**	Me	C(CH_3_)_3_	COOH	Et	9.093	8.343
**D4**	Me	OC(CH_3_)_3_	SO_3_H	Et	10.845	8.064
**D5**	Me	CH_2_OOCCH_3_	SO_3_H	Et	9.604	8.835
**D6**	Me	C(CH_3_)_3_	SO_3_H	Et	9.171	8.970
**D7**	H	C(CH_3_)_3_	COOH	Me	9.056	8.885
**D8**	Me	OC(CH_3_)_3_	NO_2_	Et	9.210	8.667
**D9**	Me	OCH(CH_3_)_2_	NO_2_	Et	9.168	8.638
**D10**	Me	CH_2_OOCCH_3_	NO_2_	Et	9.792	8.668
**D11**	Me	OC(CH_3_)_3_	CN	Et	9.129	9.052
**D12**	Me	OCH(CH_3_)_2_	CN	Et	9.288	8.966
**D13**	Me	C(CH_3_)_3_	CN	Et	9.651	9.121
**D14**	H	C(CH_3_)_3_	CF_3_	Et	9.245	9.408
**D15**	H	OC(CH_3_)_3_	CF_3_	Et	8.857	9.328
**D16**	Me	C(CH_3_)_3_	CF_3_	Et	9.430	9.250
